# Transcriptome Characterization for Non-Model Endangered Lycaenids, *Protantigius superans* and *Spindasis takanosis*, Using Illumina HiSeq 2500 Sequencing

**DOI:** 10.3390/ijms161226213

**Published:** 2015-12-16

**Authors:** Bharat Bhusan Patnaik, Hee-Ju Hwang, Se Won Kang, So Young Park, Tae Hun Wang, Eun Bi Park, Jong Min Chung, Dae Kwon Song, Changmu Kim, Soonok Kim, Jae Bong Lee, Heon Cheon Jeong, Hong Seog Park, Yeon Soo Han, Yong Seok Lee

**Affiliations:** 1 Department of Life Science and Biotechnology, College of Natural Sciences, Soonchunhyang University, 22 Soonchunhyangro, Shinchang-myeon, Asan, Chungcheongnam-do 31538, Korea; drbharatbhusan4@gmail.com (B.B.P.); hwamux@naver.com (H.-J.H.); bioksw@naver.com (S.W.K.); cindysory@naver.com (S.Y.P.); wth14@naver.com (T.H.W.); eunbi9154@naver.com (E.B.P.); jong6922@daum.net (J.M.C.); elegangce@naver.com (D.K.S.); 2 Trident School of Biotech Sciences, Trident Academy of Creative Technology (TACT), Chandaka Industrial Estate, Chandrasekharpur, Bhubaneswar, Odisha 751024, India; 3 National Institute of Biological Resources, 42, Hwangyeong-ro, Seo-gu, Incheon 22689, Korea; snubull@korea.kr (C.K.); sokim90@korea.kr (S.K.); 4 Korea Zoonosis Research Institute (KOZRI), Chonbuk National University, 820-120 Hana-ro, Iksan, Jeollabuk-do 54528, Korea; jblee@jbnu.ac.kr; 5 Hampyeong County Insect Institute, Hampyeong County Agricultural Technology Center, 90, Hakgyohwasan-gil, Hakgyo-myeon, Hampyeong-gun, Jeollanan-do 57158, Korea; heoncheonj@hanmail.net; 6 Research Institute, GnC BIO Co., LTD. 621-6 Banseok-dong, Yuseong-gu, Daejeon 34069, Korea; 5022daniel@gmail.com; 7 College of Agriculture and Life Science, Chonnam National University 77 Yongbong-ro, Buk-gu, Gwangju 61186, Korea; hanys@chonnam.ac.kr

**Keywords:** *Protantigius superans*, *Spindasis takanosis*, endangered species, transcriptome, Illumina sequencing, BLAST2GO, SSRs (simple sequence repeats)

## Abstract

The Lycaenidae butterflies, *Protantigius superans* and *Spindasis takanosis*, are endangered insects in Korea known for their symbiotic association with ants. However, necessary genomic and transcriptomics data are lacking in these species, limiting conservation efforts. In this study, the *P. superans* and *S. takanosis* transcriptomes were deciphered using Illumina HiSeq 2500 sequencing. The *P. superans* and *S. takanosis* transcriptome data included a total of 254,340,693 and 245,110,582 clean reads assembled into 159,074 and 170,449 contigs and 107,950 and 121,140 unigenes, respectively. BLASTX hits (*E*-value of 1.0 × 10^−5^) against the known protein databases annotated a total of 46,754 and 51,908 transcripts for *P. superans* and *S. takanosis*. Approximately 41.25% and 38.68% of the unigenes for *P. superans* and *S. takanosis* found homologous sequences in Protostome DB (PANM-DB). BLAST2GO analysis confirmed 18,611 unigenes representing Gene Ontology (GO) terms and a total of 5259 unigenes assigned to 116 pathways for *P. superans*. For *S. takanosis*, a total of 6697 unigenes were assigned to 119 pathways using the Kyoto Encyclopedia of Genes and Genomes (KEGG) pathway database. Additionally, 382,164 and 390,516 Simple Sequence Repeats (SSRs) were compiled from the unigenes of *P. superans* and *S. takanosis*, respectively. This is the first report to record new genes and their utilization for conservation of lycaenid species population and as a reference information for closely related species.

## 1. Introduction

Butterflies form an invincible part of the Earth’s rich biodiversity and are considered as quality-of-life indicators. Butterflies accord aesthetic value, are part of our natural heritage, and are portrayed as a symbol of beauty, peace or freedom. They are also of immense scientific value and have been used in diverse areas of biological research including pest control, population dynamics, biodiversity conservation, evolution, and genetics. Lately, they have been studied in the context of climate change and global warming. This is not surprising, as populations of many butterfly species have declined and many show substantial changes in their distribution. The primary cause for the vulnerability of butterfly species includes the damage to habitations due to human activities, agricultural activities and air pollution [1,2]. The consistent decline in butterfly populations has prompted ecologists to engage in species and community conservation initiatives that explore themes such as the evolutionary origins of butterfly diversity, population dynamics and threats (parasitism, predation and human impacts). Other scientific strategies used for the conservation assessment of butterfly populations around the globe include expressed sequence information, which is whole genome or transcriptome sequencing to characterize genes for positive selection of the species.

In the context of the declining butterfly populations, a Red List assessment is imperative as it addresses the likelihood and predisposition of the species in becoming extinct in their natural environments and hence promotes the prioritization and strict assessment of conservation programs [1]. The Red List of butterflies has been published on different scales and from different countries following the guidelines of the International Union for Conservation of Nature and Natural Resources (IUCN) [1,3,4]. In the Korean peninsula, an exhaustive investigation on the endangered butterfly populations was conducted to identify critically endangered, endangered, and vulnerable species to understand the causes of decline over time [5]. Subsequent to this, a Red List assessment was conducted in Korea using the IUCN Red List Categories and Criteria (version 3.1) and IUCN Red List Categories and Criteria (version 8.0), that classified the status of animals and plant species into several categories [6]. From these assessments, we summarized that a majority of threatened butterflies (classified as Critically Endangered, Endangered, and Vulnerable) belonged to Family Lycaenidae and Nymphalidae of the Lepidopteran taxon. The Lycaenidae Family of butterflies (gossamer-winged butterflies) represents around 40% of all known butterfly species. Generally, the butterfly species from the family (about 75% of species) show a mutualistic or commensal relationship with ants in nature, but few have evolved into parasites of ants [7,8]. Many such ant-parasitic Lycaenid species are highly endangered and are the prime focus of insect conservation biology [9]. A total of 15 Lycaenid species have been included in the Korean Red List of Threatened Species (2014). The population of these Lycaenid species have shown a decline due to climate change and loss of symbiotic ants. An extinction in host ant species is detrimental to the existence of such associated ant-parasitic Lycaenid butterflies, hence a conservation of the former is also a priority to protect the latter in the Korean peninsula [10].

The Lycaenidae family species *Spindasis takanosis* and *Protantigius superans* were listed as endangered in Korea by a Ministry of Environment report in 2005 [11]. These Lycaenid have been classified as Level 2 species and are vulnerable to face extinction unless threatening factors are eliminated or mitigated. *Spindasis takanosis* (Matsumara) has been reported from Gyunggi-do, Gangwon-do, Chungchungnam-do and Jeollanam-do provinces of Korea. The population of this species has declined alarmingly due to the loss of forests and symbiotic ants such as *Crematogaster matsumurai* [12]. *S. takanosis* belongs to the majority non-parasitic species of Lycaenids that are symbionts of *C. matsumurai* [8]. *Protantigius superans* was not included as a threatened butterfly species in an earlier study [5], but it was included as a vulnerable species by the Korean Red List assessment (2014). The ant-parasitism status of *P. superans* has not been reported. In fact, there are only scattered occurrences of ant-parasitism among the Lycaenidae species, suggesting an independent evolution of such interactions. The major barrier for the conservation efforts even after the legalization of the Korean Endangered Species Act in 2005, was the lack of data that determined the genetics, ecology and distribution of the species. As an initiative towards preserving the gene pool of the Lycaenid species *S. takanosis* and *P. superans*, the genomic and RNA sequence information initiative was considered as a foolproof strategy, more so after the completion of the mitogenome sequencing for the species [13,14]. The need for conservation genomics initiatives towards biodiversity assessment and management has been advocated, especially with the rapid evolution of Next-Generation Sequencing (NGS) platforms [15,16]. The NGS enables the derivation of a global gene or RNA expression profile that could lead to the discovery of genetic markers such as Simple Sequence Repeats (SSRs) and Single Nucleotide Polymorphisms (SNPs), and candidate transcripts as markers for ecological fitness, quantitative trait loci (QTL) and so on [17,18].

The transcriptome sequencing of butterfly species using NGS has provided with clues towards understanding the population genetic structure and setting conservation goals and priorities. A case in this initiative pertains to the rapidly declining populations of the marsh fritillary butterfly, *Euphydryas aurinia*, wherein next-generation 454-pyrosequencing characterized seven novel microsatellite loci [19]. Other 454-sequencing data has established the transcriptome of the Glanville fritillary butterfly (*Melitaea cinxia*) appropriately, with the discovery of large number of SNPs [18]. The phylogeography of the Karner blue butterfly, belonging to the genus Lycaeides, using 454-pyrosequencing has also been reported [20]. Lately, Illumina sequencing has been extremely successful in the identification of SSRs in endangered specimens within and across taxonomic groups [21,22]. Most notably, the genome assembly of the Monarch butterfly involved the use of Illumina paired-end sequencing [23]. We have used the Illumina HiSeq 2500 NGS technology to characterize the transcriptome of endangered Lycaenids *S. takanosis* and *P. superans* and annotated the genomic resources from the species for the mechanistic dissection of ecologically relevant traits. This study also bridges the gap between the genomic sequence information of model organisms *vs.* non-model species that are essentially the viable targets of biodiversity conservation and phylogenetics.

## 2. Results and Discussion

### 2.1. Transcriptome Analysis

**Figure 1:**
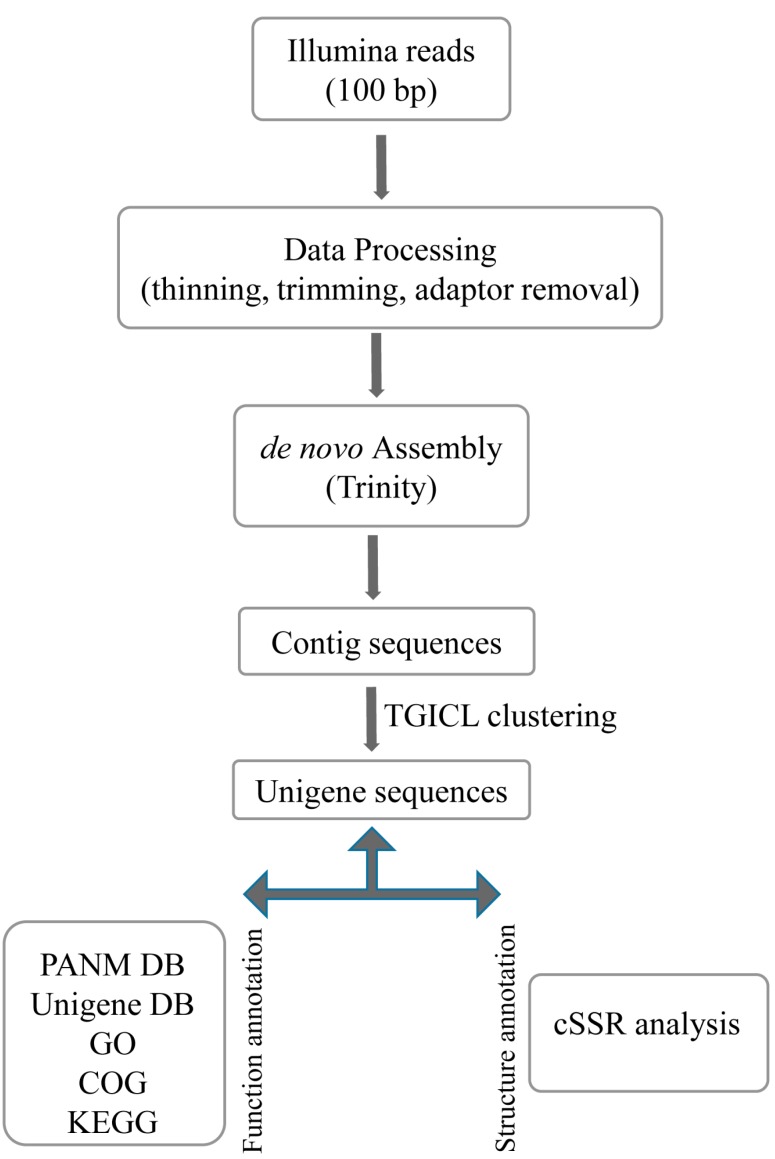
Schematic work-flow followed for the transcriptome analysis of Lycaenid butterflies, *Protantigius superans* and *Spindasis takanosis*.

In order to obtain the transcriptomes of the endangered Lycaenid butterflies *S. takanosis* and *P. superans*, a cDNA library was constructed from the RNA isolated from the whole body of adult insects and sequenced on the Illumina HiSeq 2500 NGS platform. The transcriptome assembly and analysis work-flow has been depicted in Figure 1.

The Illumina HiSeq 2500 sequencing of *P. superans* generated a total of 258,875,070 raw reads (32,618,258,820 bases) with a mean length of 126 bp. The filtering of reads based on quality parameters resulted in the discard of 0.32% of bases to get a paired-end profile of 32,514,410,974 bases with an average length of 125.6 bp. After stringent quality assessment, a total of 254,340,693 clean reads (Q_20_ ~99% and percent of unknown nucleotide is 0%) were obtained, which represents 98.25% of the obtained raw reads. The mean length and the N50 length of the obtained clean reads were 124.3 bp and 126 bp, respectively, with GC% (or guanine-cytosine content) of 39.81%. In the case of *S. takanosis*, the transcriptome generated a total of 249,312,792 raw reads (31,413,411,792 bases) with a mean length of 126 bp. Adapter trimming led to a discard of 0.35% of the total base pairs processed, which, after stringent quality assessment, generated a total of 245,110,582 clean reads (30,515,812,866 bases). The mean length, N50 length, and GC% of the obtained clean reads were 124.5, 126, and 41.96%, respectively. The summary of the read processing analysis based on quality parameters is shown in Table S1. The sequence reads generated from the transcriptome sequencing of *P. superans* and *S. takanosis* have been submitted to GenBank Sequence Read Archive (SRA) at National Center for Biotechnology Information (NCBI) under accession numbers SRP063812 and SRP063813, respectively.

**Figure 2:**
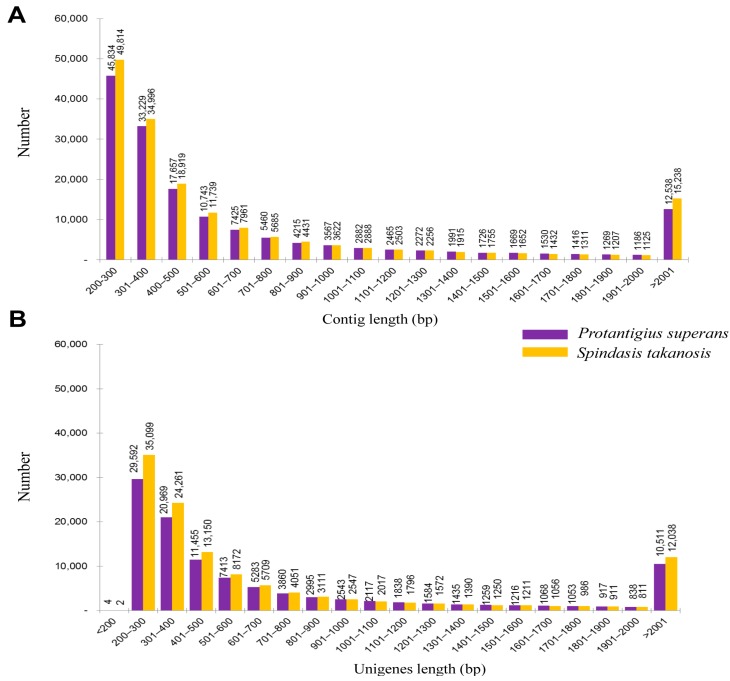
Size distribution of contig (**A**); and unigenes (**B**) after assembly and clustering of the quality reads from the transcriptomes of *P. superans* and *S. takanosis*.

The processed high quality reads were assembled using the Trinity program which uses three sequential software modules, namely Inchworm, Chrysalis, and Butterfly, for *de novo* transcriptome assembly [24]. Trinity is an exclusive program for assembling transcript sequences from Illumina transcriptome data and scores over other *de novo* transcriptome algorithms developed including SOAPdenovo-Trans [25], Trans-ABySS [26], and Oases [27]. With Trinity, a total of 159,074 contigs were assembled for the *P. superans* transcriptome, with N50 (contig length such that equal or longer contigs amount to half of the total assembly length) and mean length of 1220 bp and 746.3 bp, respectively. A significant proportion of the assembled contigs (39.28%) were ≥500 bp with the longest contig size of 15,152 bp. A total of 170,449 contigs were assembled for the *S. takanosis* transcriptome, with N50 and mean length of 1372 bp and 786.4 bp, respectively. Out of the total assembled contigs, 66,844 contigs (39.21%) were ≥500 bp with the longest contig size of 16,820 bp. The size distribution of the assembled contigs for the *P. superans* and *S. takanosis* transcriptomes have been shown in Figure 2A. It is evident that the proportion of short contigs and contigs over 1 kb were high in our datasets. Also, the contig N50 value was found higher in the Lycaenid butterfly transcriptome compared to N50s obtained from transcriptome assemblies of distinct insects [28,29,30]. The contigs were finally clustered to a total of 107,950 unigenes with 89,022,313 bases for *P. superans* and 121,140 unigenes with 100,232,710 bases for *S. takanosis*. For *P. superans*, the N50 and mean length of unigenes were 1452 and 824.7 bp, respectively, with a GC% of 38.46%. Among these unigenes, 29,596 (27.42%) had a size of no more than 300 bp, 54,518 (50.50%) were in the sizes of 301–1000 bp, 13,325 (12.34%) were of lengths in between 1001 and 2000 bp, and 10,511 (9.74%) were over 2000 bp. For *S. takanosis*, the N50 and mean length of unigene sequences were 1537 and 827.4 bp, respectively, with a GC% of 38.68%. Among these unigenes, 35,101 (28.97%) had a size of ≤300 bp, 61,001 (50.36%) were in the size range of 301–1000 bp, 13,000 (10.73%) were of lengths 1001–2000 bp, and 12,038 (9.93%) were over 2000 bp. The size distribution of assembled unigenes for both the Lycaenid butterflies are shown in Figure 2B. A summary of the *P. superans* and *S. takanosis* transcriptomes depicting the datasets obtained after the processing of raw reads, Trinity *de novo* assembly, and TGIR Gene Indices Clustering Tool (TGICL) clustering are depicted in Table 1. The transcriptome sequence and assembly efficiency was better in case of *S. takanosis* as it resulted in greater number of transcripts from a lesser number of raw read sequences.

**Table 1:** Summary of transcriptome assembly after Illumina HiSeq 2500 sequencing of Lycaenid butterflies *Spindasis takanosis* and *Protantigius superans*.

Assembly Features	*Spindasis takanosis*	*Protantigius superans*
**Raw Reads**		
Number of sequences	249,312,792	258,875,070
Number of bases	31,413,411,792	32,618,258,820
Mean length (bp)	126	126
**Clean reads**		
Number of sequences	245,110,582	254,340,693
Number of bases	30,515,812,866	31,607,701,940
Mean length (bp)	124.5	124.3
N50 length (bp)	126	126
GC%	41.96	39.81
High-quality reads (%)	98.31 (sequences), 97.14 (bases)	98.25 (sequences), 96.90 (bases)
Number of reads discarded (%)	1.69 (sequences), 2.86 (bases)	1.75 (sequence), 3.1 (bases)
**Contig information**		
Total number of contig	170,449	159,074
Number of bases	134,036,728	118,721,203
Mean length of contig (bp)	786.4	746.3
N50 length of contig (bp)	1372	1220
GC% of contig	38.58	38.45
Longest contig (bp)	16,820	15,152
No. of large contigs (≥500 bp)	66,844	62,485
Unigene information-		
Total number of unigenes	121,140	107,950
Number of bases	100,232,710	89,022,313
Mean length of unigene (bp)	827.4	824.7
N50 length of unigene (bp)	1537	1452
GC% of unigene	38.68	38.46
Length ranges (bp)	124–16,820	114–17,062

### 2.2. Sequence Annotation

The assembled unigenes of *P. superans* and *S. takanosis* were used as query sequences and blasted (BLASTX search; *E*-value ≤ 1.0 ×10^−5^) against various protein databases, including a locally curable Protostome DB (PANM-DB), Unigene and EuKaryotic Orthologous Groups (KOG) databases. Significant matches were found for the assembled unigenes with the subject sequences in PANM-DB with 44,529 (41.25%) and 46,852 (38.68%) unigenes of *P. superans* and *S. takanosis* recovering the BLAST results. Similarly, a total of 15,331 (14.2%) unigenes of *P. superans* and 22,124 (18.26%) unigenes of *S. takanosis* found homology to sequences in the Unigene database. Roughly, 18,511 (17.14%) and 24,603 (20.31%) unigenes of *P. superans* and *S. takanosis* show BLASTX hits in the KOG database. The sequence-based annotation of unigenes from the *P. superans* and *S. takanosis* transcriptomes are shown in Table 2.

**Table 2:** Sequence annotation of unigenes assembled from the *Protantigius superans* and *Spindasis takanosis* transcriptomes.

Databases	All Annotated Transcripts		≤300 bp		300–1000 bp		≥1000 bp	
	*P. superans*	*S. takanosis*	*P. superans*	*S. takanosis*	*P. superans*	*S. takanosis*	*P. superans*	*S. takanosis*
PANM-DB	44,529	46,852	6272	7342	19,244	19,666	19,013	19,844
UNIGENE	15,331	22,124	1267	2751	5098	7848	8966	11,525
KOG	18,511	24,603	1273	2721	5399	7971	11,839	13,911
GO	18,661	22,275	1956	2705	6355	7566	10,350	12,004
KEGG	5259	6697	541	897	1615	2289	3103	3511
ALL	46,754	51,908	6739	8721	20,557	22,559	19,458	20,628

**Figure 3:**
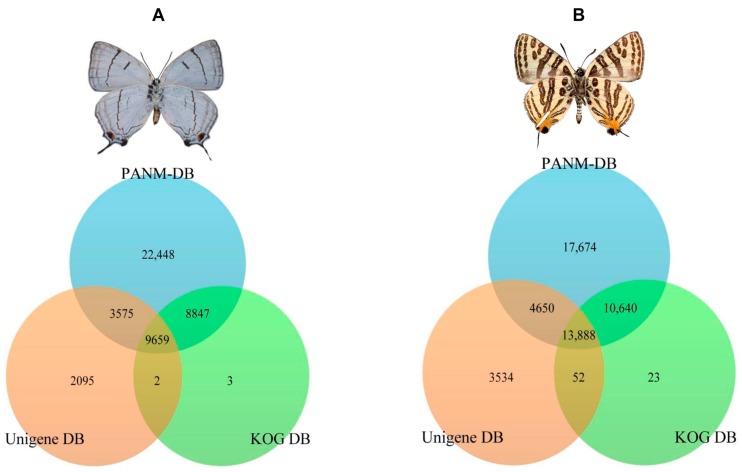
A summary depicting the annotation of *P. superans* (**A**); and *S. takanosis* (**B**) unigenes against PANM-DB, Unigene DB, and KOG DB.

We found that a significant proportion of longer unigene sequences (≥1000 bp) are circumstantial in returning a higher proportion of BLAST hits against various protein databases as compared to short-read unigenes (≤300 bp). In the case of *P. superans*, a total of 18,506 and 13,234 unigenes had common homologous matches in the PANM-DB and KOG DB and PANM-DB and Unigene DB, respectively. A total of 9659 unigene sequences overlapped within the three protein databases. Only three unigenes sequences were found exclusive to the KOG DB, whereas two sequences were uniquely shared between the KOG and Unigene DB. A total of 22,448 and 2095 sequences showed homologies exclusive to the PANM-DB and Unigene DB, respectively. The sequence annotation results for the *P. superans* butterfly are shown in Figure 3A. The unigene annotation for *S. takanosis* (Figure 3B) showed a total of 13,888 sequences overlapping the three databases. A total of 24,528, 18,538, and 13,940 unigenes recovered BLAST hits from both the PANM-DB and KOG DB, the PANM-DB and Unigene DB, and the KOG DB and Unigene DB, respectively. BLASTX was also used to search for the matches of *P. superans* and *S. takanosis* unigenes against Gene Ontology (GO) and Kyoto Encyclopedia of Genes and Genomes (KEGG) protein functional databases. A total of 18,661 (17.29%) and 5259 (4.87%) unigenes of *P. superans* found matches to protein sequences in the GO and KEGG databases, respectively (Table 2). As expected, the majority (more than 55%) of GO and KEGG annotated transcripts were ≥1000 bp. The non-annotated transcripts may also attribute to novel genes, but it is possible that most of these shorter sequences may lack a functional conserved domain and hence are missing sequence matches in the databases.

### 2.3. Homology Characteristics of Assembled Unigenes

**Figure 4:**
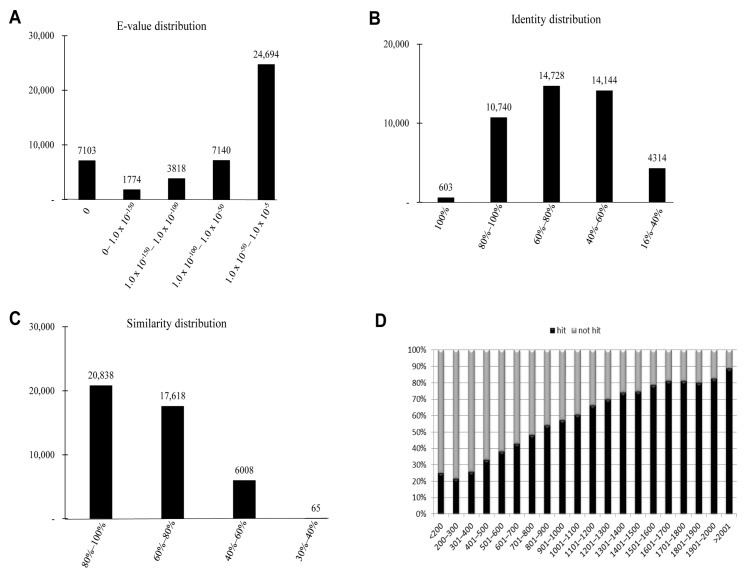
Homology search characteristics of *P. superans* assembled unigenes against PANM database. (**A**) *E*-value distribution of BLAST hits for each unigene with a cutoff value of 1.0 × 10^−5^; (**B**) Identity distribution of BLAST hits for each unigene; (**C**) Similarity distribution of BLAST hits for each unigene; (**D**) Unigene lengths with or without hits.

The characteristics of the homology search of assembled unigenes from *P. superans* recovered by BLAST hits against the PANM-DB were analyzed. We studied for homology characteristics such as the *E*-value, identity and similarity distribution (Figure 4). We have also performed the homology search of assembled unigenes against the Unigene DB (Figure S1). The *E*-value distribution revealed that a significant proportion of unigenes (24,694, 55.46%) showed significant homology to previously deposited sequences in the PANM-DB with an *E*-value ranging from 1.0 × 10^−50^ to 1.0 × 10^−5^ (Figure 4A). Identity distribution chart revealed a close distribution with 14,728 (33.08%), 14,144 (31.76%), and 10,740 (24.12%) unigenes showing identity of 60%–80%, 40%–60%, and 80%–100%, respectively. About 603 (1.35%) unigenes showed an identity of 100% to match subject sequences in the PANM-DB (Figure 4B). According to the similarity distribution chart, 20,838 (46.80%) unigenes had a similarity of 80%–100% and 17,618 (39.57%) unigenes had a similarity of 60%–80% with the deposited sequences (Figure 4C). In addition, our results showed that the unigene hit percentage increased steadily with an increase in the length of the unigenes. Above 70% of *P. superans* unigene sequences over 1500 bp in length showed BLASTx hits to protein sequences in the PANM-DB. In contrast, only close to 20% of sequences shorter than 300 bp found a hit to homologous sequences in the database (Figure 4D). In the homology search of *P. superans* assembled sequences using the Unigene DB (Figure S1), we found a majority showing an *E*-value ranging from 1.0 × 10^−50^ to 1.0 × 10^−5^ (Figure S1A). The identity distribution plot revealed most assembled sequences showing an identity of 80%–100% (Figure S1B) to sequences in the Unigene DB. About 50% of sequences >2001 bp showed annotation hits, while none of the <200 bp sequences were annotated to sequences in the database (Figure S1C).

**Figure 5:**
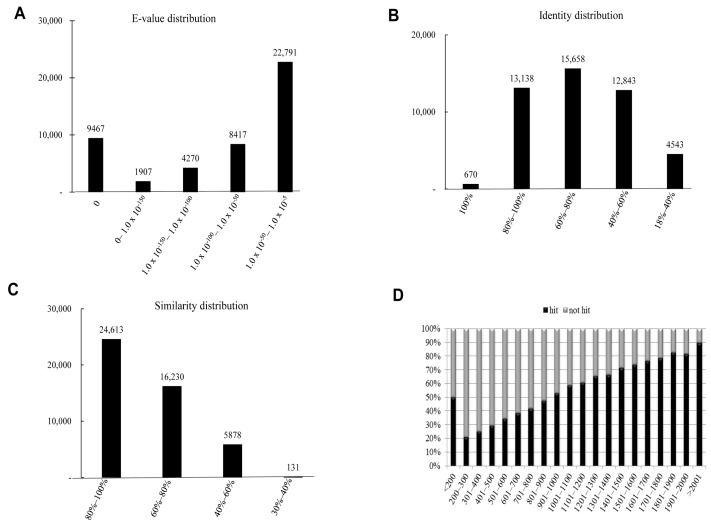
Homology search characteristics of *S. takanosis* assembled unigenes against PANM database. (**A**) *E*-value distribution of BLAST hits for each unigene with a cutoff value of 1.0 × 10^−5^; (**B**) Identity distribution of BLAST hits for each unigene; (**C**) Similarity distribution of BLAST hits for each unigene; (**D**) Unigene lengths with or without hits.

We have summarized the characteristics of the homology search of assembled unigenes from *S. takanosis* in Figure 5. The *E*-value distribution chart revealed that about 22,791 (48.64%) unigenes showed an *E*-value ranging from 1.0 × 10^−50^ to 1.0 × 10^−5^ (Figure 5A). As with *P. superans*, *S. takanosis* unigenes reveal close identity distribution with 15,658 (33.42%), 13,138 (28.04%), and 12,843 (27.41%) unigenes showing identity in the range of 60%–80%, 80%–100%, and 40%–60%, respectively. About 670 (1.43%) unigenes showed an identity of 100% to subject sequences in the PANM-DB (Figure 5B). The similarity distribution chart shows a larger share of unigenes (24,613, 52.53%) having similarity in the range of 80%–100% (Figure 5C). Unlike the *P. superans* unigenes hit percentage, almost 50% of the unigenes in *S. takanosis* with a length of less than 200 bp showed a positive hit. Following the same, the hit percentage increased by about 90% for unigenes above 2001 bp in length (Figure 5D). Homology search of *S. takanosis* assembled sequences using the Unigene DB (Figure S2) showed a majority of sequences having an *E*-value ranging from 1.0 × 10^−50^ to 1.0 × 10^−5^ (Figure S2A). A majority of sequences showed an identity of 80%–100% (Figure S2B) to sequences in the Unigene DB. Assembled sequences of >2001 bp showed about 60% annotation hits to sequences in the Unigene DB (Figure S2C). Moreover, more unigene hits were observed with the PANM-DB compared to the Unigene DB over the length of assembled sequences. This indicates that longer unigenes are more likely to get an identifiable affiliation during BLAST matches, due to the likely presence of a representative protein domain that may be hard to find in shorter sequences. More so, the longest unigenes yield BLAST hits and annotations with a higher frequency [31,32].

**Figure 6:**
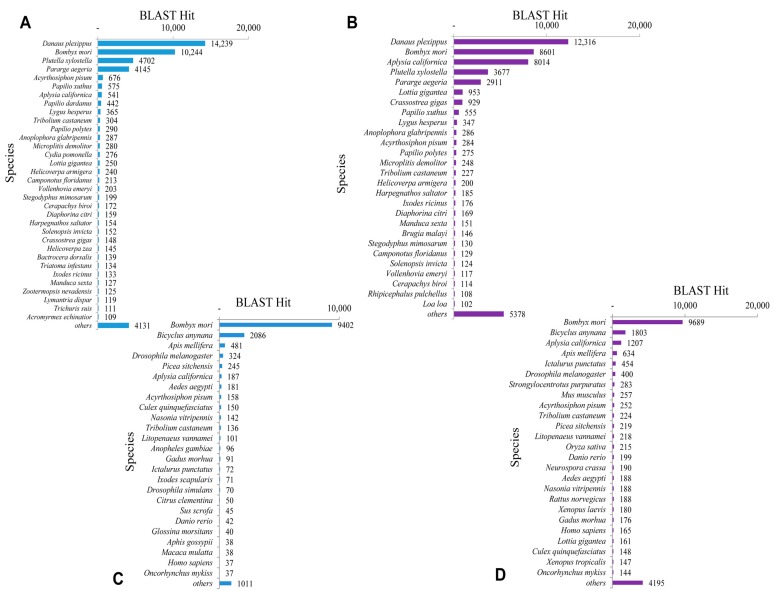
Top-hit species distribution. BLASTx top-hit species distribution of *P. superans* and *S. takanosis* against PANM-DB (**A**,**B**); and Unigene DB (**C**,**D**).

The BLASTx top-hit species distribution of unigenes matched to the PANM-DB has been shown for *P. superans* and *S. takanosis* in Figure 6A,B, respectively. In the case of both the butterfly species, the highest match was observed to *Danaus plexippus* (14,239 unigenes, 13.19%, for *P. superans*; and 12,316 unigenes, 10.17%, for *S. takanosis*), followed by *Bombyx mori* (10,244 unigenes, 9.49%; and 8601 unigenes, 7.10%). Other species ranked high with a greater number of hits included the Arthropods such as *Plutella xylostella*, *Pararge aegeria* and the mollusk *Aplysia californica* among others with more than 1000 unigene hits. We also analyzed the top-hit species distribution of the 15,331 and 22,124 assembled sequences for *P. superans* (Figure 6C) and *S. takanosis* (Figure 6D) matched to the Unigene database. The highest BLASTx hits were shown to *Bombyx mori* with 9402 and 9689 unigenes of *P. superans* and *S. takanosis*, respectively, showing matches.

**Figure 7:**
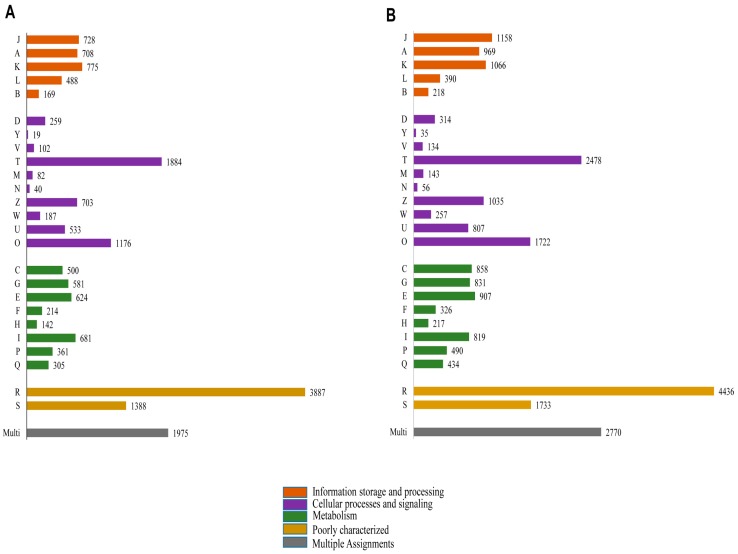
Clusters of orthologous groups’ classification (KOG) of *P. superans* (**A**); and *S. takanosis* (**B**) unigenes into four major categories of information storage and processing, cellular processes and signaling and metabolism. The code descriptions for KOG categories are as follows: J, translation, ribosomal structure, and biogenesis; A, RNA processing and modification; K, transcription; L, replication, recombination, and repair; B, chromatin structure and dynamics; D, cell cycle control, cell division, and chromosome portioning; Y, nuclear structure; V, defense mechanisms; T, signal transduction mechanisms; M, cell wall/membrane/envelope biogenesis; N, cell motility; Z, cytoskeleton; W, extracellular structures; U, intracellular trafficking, secretion, and vesicular transport; O, post-translational modification, protein turnover, and chaperones; C, energy production and conversion; G, carbohydrate transport and metabolism; E, amino acid transport and metabolism; F, nucleotide transport and metabolism; H, co-enzyme transport and metabolism; I, lipid transport and metabolism; P, inorganic ion transport and metabolism; Q, secondary metabolites biosynthesis, transport and catabolism; R, general function prediction only; S, unknown function; Multi, more than one classified function.

Functional prediction and classification of the butterfly unigenes were achieved by a search against the KOG database (Figure 7). A total of 18,511 (17.15% of total unigenes) and 24,603 (20.31% of total unigenes) unigenes of *P. superans* (Figure 7A) and *S. takanosis* (Figure 7B), respectively were ascribed functions under 25 categories arranged to four main functional groups. A greater proportion of unigenes were clustered to the cellular processes and signaling group (4985 unigenes for *P. superans* and 6981 unigenes for *S. takanosis*), followed by the metabolism group (3408 for *P. superans* and 4882 for *S. takanosis*) and the information storage and processing group (2868 for *P. superans* and 3801 for *S. takanosis*). Within the cellular processes and signaling group, most of the unigenes were classified under the signal transduction process category followed by the post-translational modification, protein turnover and chaperone categories. Furthermore, a significant fraction of unigenes remained poorly characterized (5275 unigenes for *P. superans* and 6169 unigenes for *S. takanosis*).

### 2.4. Functional Annotation Using GO and KEGG

**Figure 8:**
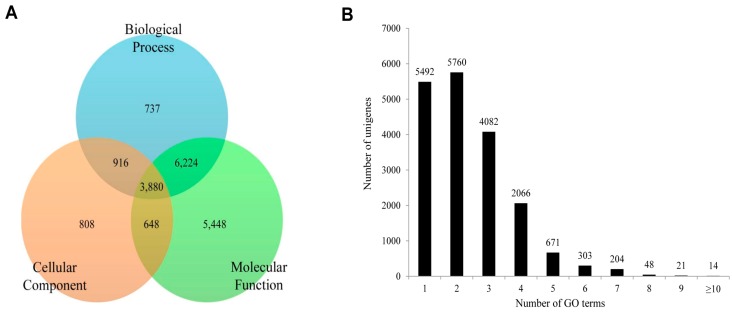
The functional distribution of the assembled unigenes of *Protantigius superans* by gene ontology assignment. (**A**) The distribution of the GO-annotated unigenes to biological process, cellular components and molecular function categories; (**B**) The number of GO term annotations ascribed to each unigene.

To functionally classify *P. superans* and *S. takanosis* unigenes, GO terms and KEGG pathway classification were assigned to each unigene using the BLAST2GO software suite. For *P. superans*, the 18,661 unigenes annotated from the total unigene profile were allocated one or more GO terms based on sequence similarity. A total of 89,289 unigene sequences were without GO terms. The GO annotated unigenes were functionally classified into three broad categories such as biological process, cellular component, and molecular function. A summary of *P. superans* unigenes and GO terms have been shown in Figure 8. The molecular function category was assigned 16,200 unigenes, followed by biological process with 11,757, and cellular components with 6252 unigenes. Of these unigenes, 3880 showed functional attributes shared within the three main categories. Additionally, 5448, 808 and 737 unigenes were found uniquely attached to the molecular function, cellular component, and biological process categories, respectively (Figure 8A). As shown in Figure 8B, a significant number of unigenes were represented by more than a single GO term. Only 5492 (29.43%) unigenes were ascribed to one GO term with most unigenes (5760, 30.87%) represented by two GO terms.

**Figure 9:**
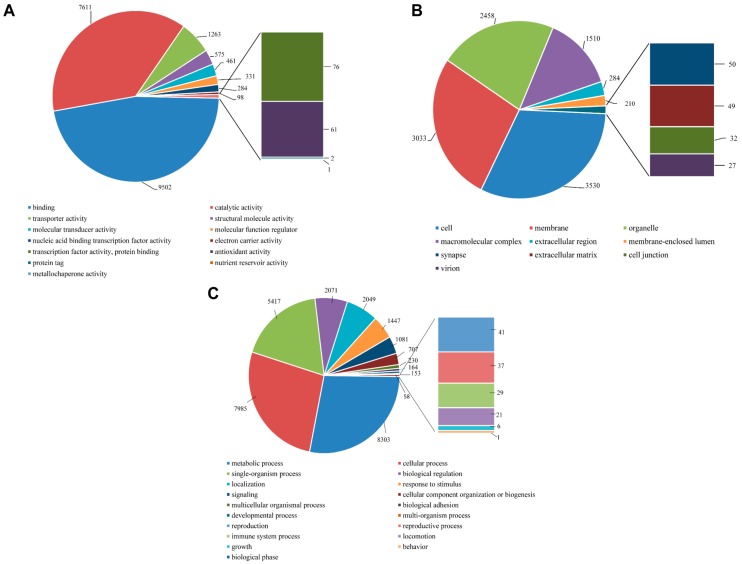
Gene ontology analysis of *P. superans* transcriptome using BLAST2GO. The number of unigenes assigned to the sub-categories under three major categories of molecular function (**A**); cellular components (**B**); and biological processes (**C**). All data are presented at level 2 GO categorization.

All unigene sequences were classified into molecular function, cellular components, and biological process level 2. Within the molecular function category, the unigenes were further classified to 13 sub-categories, out of which a majority were represented under binding (9502 unigenes, 46.89%), catalytic activity (7611, 37.56%), and transporter activity (1263, 6.23%). About 61 sequences represented the antioxidant activity with very few transcripts assigned to protein tag, nutrient reservoir activity, and metallochaperone activity (Figure 9A). Within the cellular components category, the majority of unigenes represented cell (3530, 31.57%), membrane (3033, 27.12%), organelle (2458, 21.98%) and macromolecular complex (1510, 13.50%). Few unigenes also represented synapse (50, 0.45%) and extracellular matrix (49, 0.44%) (Figure 9B). For the biological process category, there were 19 sub-categories, and metabolic process (8303, 27.86%), cellular process (7985, 26.79%) and single-organism process (18.18%) were the predominant GO groups. A smaller proportion of unigenes also fell under response to stimulus (1447, 4.86%), signaling (1081, 3.63%), reproduction (41, 0.14%), reproductive process (37, 0.12%), and immune system process (29, 0.10%) (Figure 9C).

**Figure 10:**
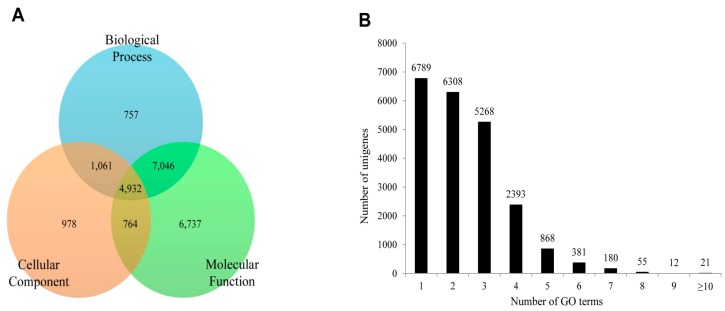
The functional distribution of the assembled unigenes of *Spindasis takanosis* by gene ontology assignment. (**A**) The distribution of the GO-annotated unigenes to biological processes, cellular components and molecular function categories; (**B**) The number of GO term annotations ascribed to each unigene.

In the case of *S. takanosis*, 22,275 unigenes (18.39% of assembled unigenes) were assigned one or more GO terms, while 98,865 unigenes were without GO terms. The GO distribution for *S. takanosis* unigenes assigned 16,200 terms to the molecular functions category, followed by 11,757 terms to the biological process and 6252 terms to the cellular components categories (Figure 10A). A total of 6737, 978, and 757 unigenes were found exclusive to molecular function, cellular components, and biological process categories, respectively. About 4932 unigenes showed functional attributes shared between the three major categories of GO terms. As with *P. superans*, a greater number *S. takanosis* unigenes (15,486) also got represented by more than one GO term. Only 6789 (30.48%) unigenes showed homology to a single GO term (Figure 10B).

**Figure 11:**
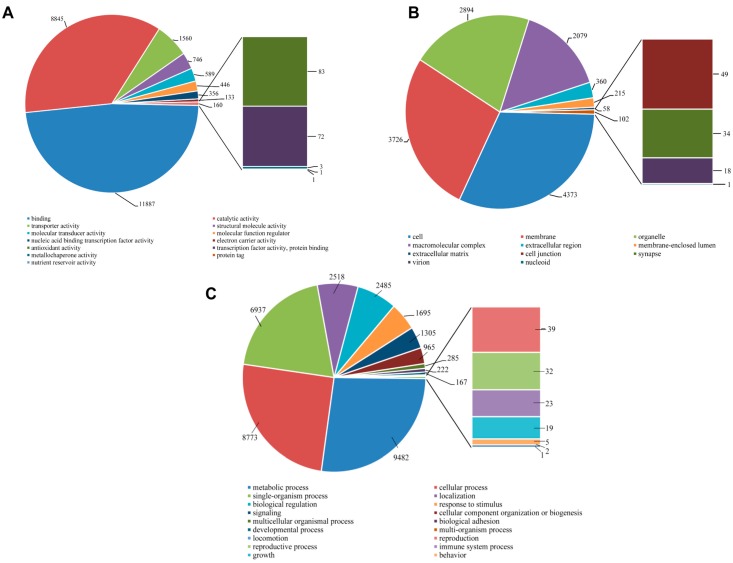
Gene ontology analysis of *S. takanosis* transcriptome using BLAST2GO. The number of unigenes assigned to the sub-categories under three major categories of molecular function (**A**); cellular components (**B**); and biological processes (**C**). All data are presented at level 2 GO categorization.

The number of *S. takanosis* unigenes ascribed to various sub-categories under the three major categories of GO terms (level 2) are shown in Figure 11. Under the molecular function category, binding (11,887 unigenes, 48.08%) and catalytic activity (8845 unigenes, 35.78%) were majorly represented. A total of 83 unigene sequences were ascribed to antioxidant activity with only three transcripts linked to metallochaperone activity (Figure 11A). Regarding the cellular components category, a high proportion of unigenes were ascribed to cell (4373 unigenes, 31.67%), membrane (3726, 26.99%), organelle (2894, 20.96%), and macromolecular complex (2079, 15.06%) (Figure 11B). Out of the 35,050 unigene hit to the biological process category, 20 sub-categories were represented. A high proportion of sequences showed homology to metabolic process (9482 unigenes, 27.05%), cellular process (8773, 25.03%) and single-organismal process (6937 unigenes, 19.79%). A total of 1305, 39, 32, and 23 unigene sequences fell under signaling, reproduction, reproductive process, and immune system process (Figure 11C). In discussing the dominant GO terms for Lycaenid butterflies *P. superans* and *S. takanosis*, we find that the profiles are very similar. The prominence of the GO biological process category over the GO molecular function and cellular components categories is understood in related species [33]. Also, the dominance of metabolic and cellular process under the GO biological process category has been consistently predicted in other Lepidopteran species. GO classification for functions in the sugarcane giant borer (*Telchin licus licus*) transcriptome show over 50% of GO terms represented under metabolic and cellular processes [34]. Consistent with our observations, the most prominent GO molecular function categories include binding and catalytic activity and the most prominent GO cellular components are cell, organelle and macromolecular complex in Lepidoptera and other representative insects [33,35].

**Figure 12:**
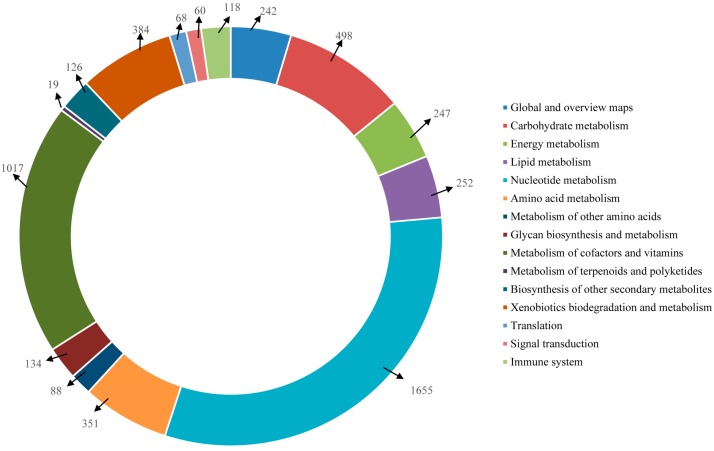
KEGG pathway assignment for *P. superans* transcriptome.

Furthermore, the unigenes were searched against the KEGG database for the identification of biological pathways active in the Lycaenid butterflies under investigation. In *P. superans*, a total of 5259 unigenes were assigned to 116 pathways. Among them, 709 enzymes were assigned to these pathways. The number of unigenes assigned to the main pathways have been presented in Figure 12. The unigenes predominantly fall into the metabolism (5013 unigenes, 95.32%) group, followed by the organismal systems (118 unigenes, 2.24%), genetic information processing (68 unigenes, 1.29%), and environmental information processing (60 unigenes, 1.14%) groups. Among the metabolism group, the majority of the unigenes were ascribed to the nucleotide metabolism sub-group (1655, 31.47%) followed by the metabolism of co-factors and vitamins (1017, 19.34%). Apart from the metabolism group, the unigenes exclusively fell under the translation sub-group for the genetic information processing group, the signal transduction sub-group for the environmental information processing group and the immune system sub-group for the organismal systems group.

**Figure 13:**
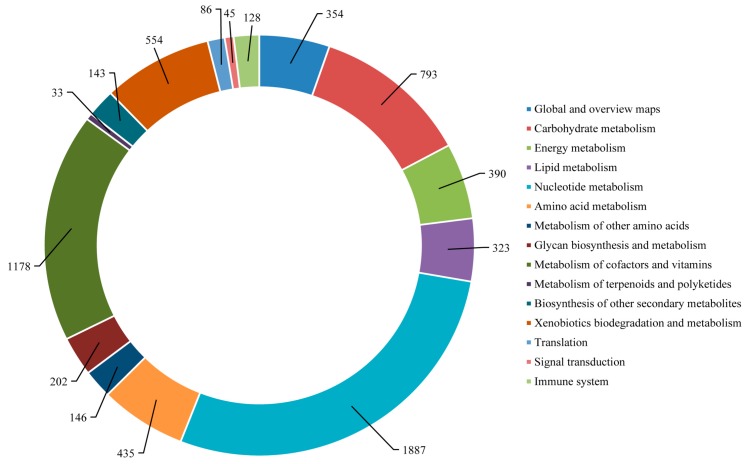
KEGG pathway assignment for *S. takanosis* transcriptome.

The mapping of *S. takanosis* annotated unigenes to typical KEGG pathways identified a total of 6697 assembled sequences assigned to 119 pathways. Among them, 800 enzymes were assigned to these pathways. A total of 6438 (96.13%) unigenes belonged to the metabolism pathway, out of which 1887 unigenes fell under nucleotide metabolism and 1178 unigenes fell under the metabolism of cofactors and vitamins sub-group. A total of 128 unigenes (1.91%) were represented under the immune system pathway under the category of organismal systems. A total of 86 and 45 unigenes fell under the translation and signal transduction pathways of the genetic information processing and environmental information processing categories, respectively (Figure 13). The KEGG pathways identified in *S. takanosis* but not in *P. superans* were chlorocyclohexane and chlorobenzene degradation, flavone and flavonol biosynthesis, fluorobenzoate degradation and steroid degradation, while the betalain biosynthesis pathway was observed in only *P. superans*.

The GO classification, in a stricter sense, does not mean evidence of functionality; instead, it only suggests that a unigene sequence can be grouped to those of known (or predicted) function. An analysis to consider is the evidence code associated with each GO term. We find that a majority of GO terms (above 99% in both the sequenced Lycaenids) represented in our study are assigned the code “IEA” (inferred from electronic annotation), which are not manually curated and probably may contain more false positives. This is true, as out of the over 16 million GO annotations as of October 2007, 15,687,382 are in fact computationally derived IEA codes [36]. Hence, in the discussion of GO term annotations for the study, we emphasize that not all GO terms are of equal validity and, based on this, the interpretations of unigenes relate only to predicted function. Additionally, we presume at this point that, using BLASTx, unigene sequences are found to share homology with known pathway genes in the KEGG database. In addition, it is critical to study both the partial and full-length unigene sequences at the functional level for major applications of transcriptome sequencing.

### 2.5. Protein Domain Analysis

InterProScan searches were conducted on the identified 107,950 *P. superans* unigenes by BLAST2GO. We discovered a total of 154,298 protein domains that include a maximum of 10,978 C_2_H_2_-like zinc finger domains. A summary of top 40 domains predicted in the *P. superans* transcriptome has been shown in Table S2. The notable conserved protein domains included the C_2_H_2_-like zinc finger, protein kinase, WD40 (also known as WD or β-transducin repeats), ABC-transporter, EGF-like (epidermal growth factor-like), Immunoglobulin-like, and fibronectin type-III domains. Among the enlisted top 40, we also found cytochrome P450, insect cuticle protein, G protein-coupled receptor, UDP-glucosyltransferase (Uridine diphosphate-glucosyltransferase), and major facilitator superfamily domains.

As with *P. superans*, a protein domain classification of *S. takanosis* transcripts was identified using BLAST2GO with the top 40 InterPro domains represented in Table S3. With the notable presence of the C_2_H_2_-like zinc finger domain, WD40 repeat domain, Armadillo-like helical domain, and cytochrome P450 domain, as also observed with *P. superans*, some other functional domains were characteristic of *S. takanosis* unigenes.

The C-type lectin domain, cadherin domain and the thioredoxin-like fold domain showed significant *S. takanosis* unigene hits. The C-type lectin domain proteins are known to be encoded by a number of genes in insects with strategic functions in the regulation of antimicrobial activity, proPO activation, and other associated immune functions [37,38].

The protein kinases and zinc finger domains show conspicuous presence in other invertebrate transcriptomes as these process cellular functions including survival, differentiation and apoptosis [31,39,40]. The transcriptome abundant and conserved C_2_H_2_-like zinc finger domains exists in proteins as multiple tandem pairs of zinc fingers or tandem arrays of three or more zinc fingers, and hence are often represented by few proteins in surveyed species. These proteins are most likely DNA-binding transcription factors but can also bind to RNA and other protein targets [41,42]. The presence of WD40 repeat and Armadillo-like helical domains in 172 and 106 unigenes of *P. superans* is consistent with similar analysis in insects and crustaceans [43,44]. The immunoglobulin and fibronectin type-III domains are basically involved in cell-signaling mechanisms related to cellular processes and immunity [31,45]. While cytochrome P450 genes are predominantly involved in the metabolism of xenobiotics in mollusks, polychaete and crustaceans, these sequences are categorized as “environmental response genes” in insects [46]. The protein domain information provides vital clues to understanding the mechanisms of cellular survival and signaling mechanisms leading to adaptation in the endangered Lycaenid butterflies, *P. superans* and *S. takanosis*.

### 2.6. Discovery of Microsatellites

**Figure 14:**
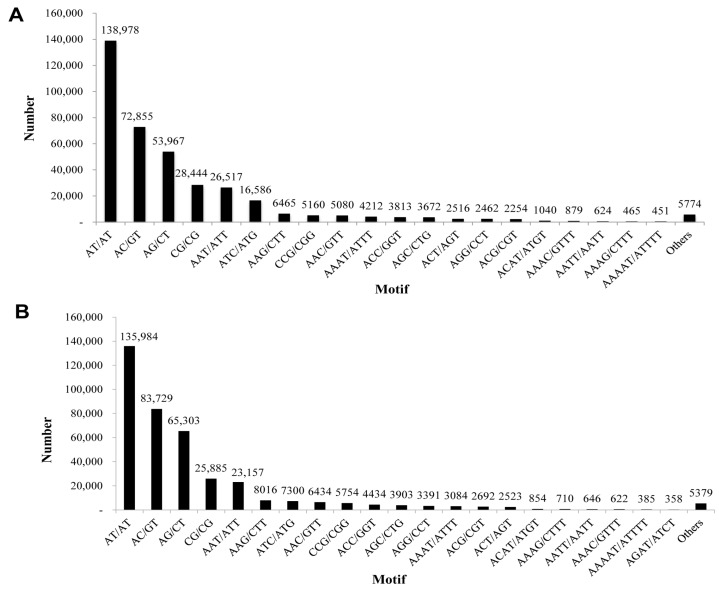
Frequency distribution of simple sequence repeats based on motif sequence types. (**A**) *P. superans*; and (**B**) *S. takanosis*.

We used the MISA (MicroSAtellite identification tool) Perl script to explore the SSR profiles in unigenes of Lycaenid butterflies *P. superans* and *S. takanosis*. In the case of *P. superans*, out of a total of 107,950 unigenes investigated, 400,330 SSRs were detected. A total of 89,877 sequences contained SSRs with 66,187 (61.31%) sequences containing more than one SSR. After eliminating the mono-nucleotide repeats (18,116 number of SSRs) and deca-nucleotide repeats (50 number of SSRs), a total of 382,164 SSRs were obtained. The di-nucleotide repeats were predominant (294,244, 76.99%) with a maximum of three tandem reiterations (254,481). In fact, three tandem repeats were the most common among the repeat motifs. The total number of di-nucleotide motifs were followed by tri- (19.5%), tetra- (2.68%), penta- (0.59%), hexa- (0.17%), hepta- (0.052%), and octa-nucleotide repeats (0.018%) (Table 3). AT/AT (138,978, 36.36%) was the most abundant motif in the SSR profile, followed by AC/GT (72,855, 19.06%), and AG/CT (53,967, 14.12%) (Figure 14A). There was a consistency observed with the tri-nucleotide repeat motifs with minor variations in the numbers. From the *S. takanosis* unigene information, we derived a total of 402,685 SSRs with 67,023 (55.33%) sequences containing more than one SSR. A total of 141,700 SSRs were present in compound form. We obtained a total of 390,516 SSRs after elimination of mono-nucleotide repeats (12,142 number of SSRs) and deca-nucleotide repeats (27 number of SSRs). The di-nucleotide repeats formed the largest group with 310,901 SSRs (79.6%) followed by the tri- (67,604), tetra- (9098), penta- (1960), hexa- (738), hepta- (148), and octa-nucleotide motifs (93) (Table 4). The most abundant repeat motifs identified were AT/AT (135,984, 34.83%), AC/GT (83,729, 21.45%), and AG/CT (65,303, 16.73%) (Figure 14B).

**Table 3:** Summary of simple sequence repeat (SSR) types in the *Protantigius superans* transcriptome.

Motif Length	Repeat Numbers																				
	3	4	5	6	7	8	9	10	11	12	13	14	15	16	17	18	19	20	≥21	Number ^#^	% ^$^
Di	254,481	30,793	5254	1495	642	425	300	190	155	142	33	70	43	33	39	29	18	20	82	294,244	76.99
Tri	61,763	8858	2514	765	332	182	29	15	14	10	7	1	9	6	3	2	2	3	10	74,525	19.5
Tetra	8967	1050	196	32	1	1	4	3	3	0	0	2	0	0	0	2	1	0	3	10,265	2.68
Penta	2024	175	32	4	5	0	0	0	0	0	0	1	0	0	0	0	0	1	0	2242	0.59
Hexa	538	82	6	9	0	2	1	0	3	0	2	0	0	0	0	0	0	0	0	643	0.17
Hepta	156	14	3	3	1	3	1	2	1	0	0	0	1	1	0	0	0	0	0	186	0.052
Octa	55	1	2	0	0	0	1	0	0	0	0	0	0	0	0	0	0	0	0	59	0.018
Total	327,984	40,973	8007	2308	981	613	336	210	176	152	42	74	53	40	42	33	21	24	95	382,164	100.00

^#^ Number of SSRs detected in the unigenes; ^$^ Relative percent of SSRs with different motif lengths among the total SSRs.

**Table 4:** Summary of simple sequence repeat (SSR) types in the *Spindasis takanosis* transcriptome.

Motif Length	Repeat Numbers																				
	3	4	5	6	7	8	9	10	11	12	13	14	15	16	17	18	19	20	≥21	Number ^#^	% ^$^
Di	272,076	31,299	4624	1078	476	291	211	149	142	140	33	46	62	57	38	30	31	17	101	310,901	79.61
Tri	59,652	5834	1249	416	215	111	16	21	16	11	4	5	4	4	8	4	3	5	26	67,604	17.31
Tetra	7734	859	295	134	10	9	6	11	8	3	8	6	7	2	2	2	1	0	1	9098	2.33
Penta	1687	185	49	6	11	6	5	3	1	4	1	0	0	0	1	0	0	1	0	1960	0.50
Hexa	613	77	5	2	3	2	1	1	3	0	1	2	1	0	1	0	0	0	0	738	0.019
Hepta	113	17	2	5	3	1	3	1	2	0	0	0	0	1	0	0	0	0	0	148	0.003
Octa	73	4	10	2	0	0	2	2	0	0	0	0	0	0	0	0	0	0	0	93	0.002
Total	341,948	38,275	6234	1643	718	420	244	188	172	158	47	59	74	64	50	36	35	23	128	390,516	100.00

^#^ Number of SSRs detected in the unigenes; ^$^ Relative percent of SSRs with different motif lengths among the total SSRs.

SSRs derived from the *Tenebrio molitor* transcriptome database also show AT as the most abundant motif and SSRs with five repeat units as the most common genetic marker [47]. Another study reported a SSR profile of 92 in the butterfly *Euphydryas editha* [48] which accumulates the rarest SSRs of Lepidopteran genomes. A thorough understanding of SSRs in the Lycaenid butterflies will be useful for the development of markers for genetic diversity assessment, gene flow characterization and conservation genomics. Additionally, SSRs from transcriptome datasets are critical for the identification of associations with functional genes and cataloguing the phenotypes [49]. With the exception of Mono-nucleotide repeat motifs that may be a result of sequencing, other repeat motifs will be suitable for polymorphic microsatellite loci identification [50,51]. A list of informative PCR primers targeting the most relevant repeat types (di-, tri-, tetra-nucleotide with a minimum of seven repeats) has been shown for *P. superans* (Table S4) and *S. takanosis* (Table S5). These sequences will be significant in further studies of genetic variation, population and conservation genomics of the species.

## 3. Experimental Section

### 3.1. Ethics Statement

For the collection of endangered Lycaenid butterflies, *S. takanosis* and *P. superans*, necessary permission was accorded from Hangang River Basin Environmental Office (Ref. No. 2014-26; 17 July 2014) and Wongju Regional Environmental Office (Ref. No. 2014-22; 30 June 2014), Korea.

### 3.2. Sample Preparation and Illumina Sequencing

The geographic origins of *P. superans* and *S. takanosis* for sequencing were Gangwon-do and Gyeonggi-do regions of Korea, respectively. A total of two individuals from each species were used for experimental purposes as per the notification of the Ministry of Environment, South Korea. Total RNA was extracted from the adults (pooled whole body samples) of *S. takanosis* and *P. superans* using Trizol Reagent (Invitrogen, Carlsbad, CA, USA) according to the manufacturer’s instructions. The processed RNA were checked for purity and integrity using Nanodrop-2000 spectrophotometer (Thermo Scientific, Wilmington, DE, USA) and the Bioanalyzer 2100 (Agilent Technologies, Santa Clara, CA, USA). The mRNA-seq library was constructed using the mRNA-seq sample preparation kit (Illumina, San Diego, CA, USA). In the process, the total RNA was treated with DNase I, and magnetic beads with Oligo(dT) to purify poly(A+) mRNA from it. The purified mRNA was fragmented using the DNA fragmentation kit (Ambion, Austin, TX, USA) prior to cDNA synthesis. The short fragments of mRNA were used to transcribe first-strand cDNA using reverse-transcriptase (Invitrogen) and random hexamer-primers. The synthesis of second-strand cDNA was accomplished using DNA polymerase I (New England BioLabs, Ipswich, MA, USA) and RNase H (Invitrogen). Subsequently, the double-stranded cDNA was end-repaired using T4 DNA polymerase, the Klenow fragment, and the T4 polynucleotide kinase (New England BioLabs). The end-repaired cDNA fragments were connected with PE (Paired-end) Adapter Oligo Mix using T4 DNA ligase (New England BioLabs) at room temperature for 15 min. The suitable fragments (200 ± 25 bp) separated on a 2% agarose gel electrophoresis matrix were paired-end sequenced on an ultra-high-throughput Illumina HiSeq 2500 sequencer. Illumina short-reads is an appropriate NGS platform for the sequencing of transcriptomes in non-model species due to its affordability and output efficiency [31,52].

### 3.3. De Novo Assembly and Annotation

The raw paired-end reads of the two Lycaenid transcriptomes (*S. takanosis* and *P. superans*) were cleaned by filtering out adapter (nucleotide length of recognized adapter ≤13 and the remaining adapter-excluded nucleotide length ≤35), repeated, and low-quality reads (phred quality score of less than 20) that may affect optimum assembly analysis and annotation. We command-line tool Cutadapt with default parameters (for paired-end reads: -a ADAPT1 -A ADAPT2; -o out1. fastq -p out2. fastq in1. fastq in2. fastq) [53] for pre-processing of raw reads. Cutadapt was selected over other popular adaptor trimmer programs [54] as it has one of the highest Mathew’s correlation coefficients (mCC)—a quality indicator for pattern recognition. The clean reads from the samples were assembled with the short reads assembling program, called Trinity (v2.0.6) [24] with 200 GB of memory and a path reinforcement distance of 50. The Trinity assembler with the default options (fastq type reads; paired read: RF; number of CPUs: eight; minimum assembled contig length of 200 bp) first assembled the reads to form longer fragments without gaps called contigs. These contigs were further assembled to unigenes (having 94% identity, 30 bp overlap) using sequence clustering software TIGR gene indices clustering tool (TGICL) [55]. After the elimination of redundant sequences, the longest transcripts were recognized as unigenes and were used for functional annotation analysis.

All the unigenes were searched against the PANM reference database (PANM-DB) [56] using the BLASTx program with an *E*-value threshold of 1.0 × 10^−5^ for the identification of functional transcripts. PANM-DB combines protein sequence data of Arthropoda, Nematoda, and Mollusks (in multi-FASTA format) downloaded from the Taxonomy browser of NCBI nr database. PANM-DB is freely downloadable from amino acid database BLAST web-interface of Malacological Society of Korea. Subsequently, the unigenes were blasted against Unigene DB [57], Eukaryotic clusters of orthologous groups (KOG) DB [58], and Kyoto Encyclopedia of Genes and Genomes (KEGG) DB [59] using BLASTX at a typical cut-off *E*-value of less than 1.0 × 10^−5^. Number of unigenes that were either unique or shared among PANM-DB, Unigene DB and KOG DB were visualized using a three-way Venn diagram plot constructed using Venny [60]. The gene ontology (GO) annotations presented represent the level 2 analysis, illustrating the predicted function of the assembled unigenes under biological process, molecular function, and cellular component category. The GO analysis was conducted using the professional BLAST2GO suite [61]. InterProScan at BLAST2GO was used to annotate the assembled unigenes with characteristic protein domains [62].

### 3.4. Identification of cSSR Markers

MicroSAtellite (MISA) [63] was used to decipher microsatellites in the unigene sequences of the Lycaenid butterflies *S. takanosis* and *P. superans*. The Simple Sequence Repeats (SSR) searches were run on default mode with detection of mono-, di-, tri-, tetra-, penta-, and hexa-nucleotide motifs, including the compound SSR (with more than one type of repeat unit). Primer pairs flanking the SSR motifs were designed using BatchPrimer3 [64] with the following criteria: primer lengths of 18–23 bases (optimum size of 21 bases), product size of 100–300 bases, Tm-50–470 °C (optimum 55 °C), and primer GC content of 30%–70%.

## 4. Conclusions

In this study, the transcriptomes of endangered Lycaenid butterflies *P. superans* and *S. takanosis* were sequenced using Illumina HiSeq 2500. A *de novo* assembly and transcript annotation approach resulted in the identification of unigenes related to functional GO categories and KEGG pathways. Furthermore, the identification of SSRs with repeat types will assist in the development of large-scale molecular markers for the species. The valuable transcriptome sequence and functional information will provide necessary cues towards the successful implementation of sustainable conservation plans for the butterfly species in their preferred habitat. The sequence information to be indexed in the databases will be the basis for an evolutionary developmental study of Lycaenidae with symbiotic ants.

## Supplementary Materials

Supplementary materials can be found at http://www.mdpi.com/1422-0067/16/12/26213/s1.

## References

[1] Fox R., Warren M.S., Brereton T.M., Roy D.B., Robinson A. (2011). A new Red List of British butterflies. Insect Conserv. Divers..

[2] Nakamura Y. (2011). Conservation of butterflies in Japan: Status, actions and strategy. J. Insect Conserv..

[3] IUCN IUCB Red List of Threatened Species. Version 2010.4. http://www.iucnredlist.org.

[4] Van Swaay C., Cuttelod A., Collins S., Maes D., Lopez Munguira M., Sasic M., Settele J., Verovnik R., Verstrael T., Warren M. (2010). European Red List of Butterflies.

[5] Choi S.W., Kim S.S. (2012). The past and current status of endangered butterflies in Korea. Entomol. Sci..

[6] National Institute of Biological Resources (2014). Korean Red List of Threatened Species.

[7] Pierce N.E., Braby M.F., Heath A., Lohman D.J., Mathew J., Rand D.B., Travassos M.A. (2002). The ecology and evolution of ant association in the Lycaenidae (Lepidoptera). Annu. Rev. Entomol..

[8] Fiedler K. (2012). The host genera of Ant-Parasitic Lycaenidae Butterflies: A Review.

[9] Thomas J.A., Simcox D.J., Clarke R.T. (2009). Successful conservation of a threatened Maculinea butterfly. Science.

[10] Bonebrake T.C., Ponisio L.C., Boggs C.L., Ehrlich P.R. (2010). More than just indicators: A review of tropical butterfly ecology and conservation. Biol. Conserv..

[11] Ministry of Environment (2005). Endangered Plants and Animals in Korea.

[12] Jang Y.J. (2007). Review on host ant of social parasitic Myrmecophiles in Korean Lycaenidae (Lepidoptera). J. Lepd. Soc. Korea.

[13] Kim I., Lee E.M., Seol K.Y., Yun E.Y., Lee Y.B., Hwang J.S., Jin B.R. (2006). The mitochondrial genome of the Korean hairstreak, *Coreana raphaelis* (Lepidoptera: Lycaenidae). Insect Mol. Biol..

[14] Kim M.J., Kang A.R., Jeong H.C., Kim K.G., Kim I. (2011). Reconstructing intraordinal relationships in Lepidoptera using mitochondrial genome data with the description of two newly sequenced lycaenids, *Spindasis takanosis* and *Protantigius superans* (Lepidoptera: Lycaenidae). Mol. Phylogenet. Evol..

[15] Allendorf F.W., Hohenlohe P.A., Luikart G. (2010). Genomics and the future of conservation genetics. Nat. Rev. Genet..

[16] Hoffman J.I., Simpson F., David P., Rijks J.M., Kuiken T., Thorne M.A., Lacy R.C., Dasmahapatra K.K. (2014). High throughput sequencing reveals inbreeding depression in a natural population. Proc. Natl. Acad. Sci. USA.

[17] Nagaraj S.H., Gasser R.B., Ranganathan S. (2007). A hitchhiker’s guide to expressed sequence tag (EST) analysis. Brief. Bioinform..

[18] Vera J.C., Wheat C.W., Fescemeyer H.W., Frilander M.J., Crawford D.L., Hanski I., Marden J.H. (2008). Rapid transcriptome characterization for a nonmodel organism using 454 pyrosequencing. Mol. Ecol..

[19] Smee M.R., Pauchet Y., Wilkinson P., Wee B., Singer M.C., French-Constant R.H., Hodgson D.J., Mikheyev A.S. (2013). Microsatellites for the Marsh Fritillary Butterfly: *De Novo* transcriptome sequencing, and a comparison with amplified length polymorphism (AFLP) markers. PLoS ONE.

[20] Gompert Z., Lucas L.K., Fordyce J.A., Forister M.L., Nice C.C. (2010). Secondary contact between *Lycaeides idas* and *L. Melissa* in the Rocky Mountains: Extensive admixture and a patchy hybrid zone. Mol. Ecol..

[21] O’Bryhim J., Chong J.P., Lance S.L., Jones K.L., Roe K.J. (2012). Development and characterization of sixteen microsatellite markers for the federally endangered species: *Leptodea leptodon* (Bivalvia: Unionidae) using paired-end Illumina shotgun sequencing. Conserv. Genet. Res..

[22] Lance S.L., Love C.N., Nunziata S.O., O’Bryhim J.R., Scott D.E., Flynn R.W., Jones K.L. (2013). 32 species validation of a new Illumina paired-end approach for the development of microsatellites. PLoS ONE.

[23] Zhan S., Merlin C., Boore J.L., Reppert S.M. (2011). The monarch butterfly genome yields insights into long-distance migration. Cell.

[24] Grabherr M.G., Haas B.J., Yassour M., Levin J.Z., Thompson D.A., Amit I., Adiconis X., Fan L., Raychowdhury R., Zeng Q. (2011). Full-length transcriptome assembly from RNA-Seq data without a reference genome. Nat. Biotechnol..

[25] Xie Y., Wu G., Tang J., Luo R., Patterson J., Liu S., Huang W., He G., Gu S., Li S. (2014). SOAPdenovo-Trans: *De novo* transcriptome assembly with short RNA-Seq reads. Bioinformatics.

[26] Birol I., Jackman S.D., Nielsen C.B., Qian J.Q., Varhol R., Stazyk G., Morin R.D., Zhao Y., Hirst M., Schein J.E. (2009). *De novo* transcriptome assembly with ABySS. Bioinformatics.

[27] Schultz M.H., Zerbino D.R., Vinqron M., Birney E. (2012). Oases: Robust *de novo* RNA-seq assembly across the dynamic range of expression levels. Bioinformatics.

[28] Jimenez-Guri E., Huerta-Cepas J., Cozzuto L., Wotton K.R., Kang H., Himmelbauer H., Roma G., Gabaldon T., Jaeger J. (2013). Comparative transcriptomics of early dipteran development. BMC Genom..

[29] Zhou X., Qian K., Tong Y., Zhu J.J., Qiu X., Zeng X. (2014). *De novo* transcriptome of the hemimetabolous German cockroach (*Blattella germanica*). PLoS ONE.

[30] Chen H., Lin L., Xie M., Zhang G., Su W. (2014). *De novo* sequencing, assembly and characterization of antennal transcriptome of *Anomala corpulenta* Motschulsky (Coleoptera: Rutelidae). PLoS ONE.

[31] Riesgo A., Andrade S.C.S., Sharma P.P., Novo M., Perez-Porro A.R., Vahtera V., Gonzalez V.L., Kawauchi G.Y., Giribet G. (2012). Comparative description of ten transcriptomes of newly sequenced invertebrates and efficiency estimation of genomic sampling in non-model taxa. Front. Zool..

[32] Wang X.J., Xu R.H., Wang R.I., Liu A.Z. (2012). Transcriptome analysis of Sacha Inchi (*Plukenetia volubilis* L.) seeds at two developmental stages. BMC Genom..

[33] Vogel H., Altincicek B., Glockner G., Vilcinskas A. (2011). A comprehensive transcriptome and immune-gene repertoire of the lepidopteran model host *Galleria mellonella*. BMC Genom..

[34] De Assis Fonseca F.C., Firmino A.A.P., de Macedo L.L.P., Coelho R.R., de Sousa Junior J.D.A., Silva-Junior O.B., Togawa R.C., Pappas Junior G.J., Brandao de Gois L.A., Mattar da Silva M.C. (2015). Sugarcane giant borer transcriptome analysis and identification of genes related to digestion. PLoS ONE.

[35] Nirmala X., Schetelig M.F., Yu F., Handler A.M. (2013). An EST database of the Caribbean fruit fly, *Anastrepha suspense* (Diptera: Tephritidae). Gene.

[36] Rhee S.Y., Wood V., Dolinski K., Draghici S. (2008). Use and misuse of the gene ontology annotations. Nat. Rev. Genet..

[37] Tanaka H., Ishibashi J., Fujita K., Nakajima Y., Sagisaka A., Tomimoto K., Suzuki N., Yoshiyama M., Kaneko Y., Iwasaki T. (2008). A genome-wide analysis of genes and gene families involved in innate immunity of *Bombyx mori*. Insect Biochem. Mol. Biol..

[38] Rao X.J., Cao X., He Y., Hu Y., Zhang X., Chen Y.R., Blissard G., Kanost M.R., Yu X.Q., Jiang H. (2015). Structural features, evolutionary relationships, and transcriptional regulation of C-type lectin-domain proteins in *Manduca sexta*. Insect Biochem. Mol. Biol..

[39] Zagrobelny M., Scheibye-Alsing K., Jensen N.B., Moller B.L., Gorodkin J., Bak S. (2009). 454 pyrosequencing based transcriptome analysis of *Zygaena filipendulae* with focus on genes involved in biosynthesis of cyanogenic glucosides. BMC Genom..

[40] Bai X.D., Mamidala P., Rajarapu S.P., Jones S.C., Mittapalli O. (2011). Transcriptomics of the bed bug (*Cimex lectularius*). PLoS ONE.

[41] Brayer K.J., Segal D.J. (2008). Keep your fingers off my DNA: Protein-protein interactions mediated by C_2_H_2_ zinc finger domains. Cell Biochem. Biophys..

[42] Seetharam A., Bai Y., Stuart G.W. (2010). A survey of well conserved families of C_2_H_2_ zinc-finger genes in Daphnia. BMC Genom..

[43] Altincicek B., Vilcinskas A. (2007). Identification of immune-related genes from an apterygote insect, the firebrat *Thermobia domestica*. Insect Biochem. Mol. Biol..

[44] Jung H., Lyons R.E., Dinh H., Hurwood D.A., McWilliam S., Mather P.B. (2011). Transcriptomics of a giant freshwater prawn (*Macrobrachium rosenbergii*): *De novo* assembly, annotation and marker discovery. PLoS ONE.

[45] Teichmann S.A., Chothia C. (2000). Immunoglobulin superfamily proteins in *Caenorhabditis elegans*. J. Mol. Biol..

[46] Meng X., Zhang Y., Bao H., Liu Z. (2015). Sequence analysis of insecticide action and detoxification-related genes in the insect pest natural enemy *Pardosa pseudoannulata*. PLoS ONE.

[47] Zhu J.Y., Wu G.X., Yang B. (2013). High-throughput discovery of SSR genetic markers in the yellow mealworm beetle, *Tenebrio molitor* (Coleoptera: Tenebrionidae), from its transcriptome database. Acta Entomol. Sin..

[48] Mikheyev A.S., Vo T., Wee B., Singer M.C., Parmesan C. (2010). Rapid microsatellite isolation from a butterfly by *de novo* transcriptome sequencing: Performance and a comparison with AFLP-derived distances. PLoS ONE.

[49] Zalapa J.E., Cuevas H., Zhu H., Steffan S., Senalik D., Zeldin E., McCown B., Harbut R., Simon P. (2012). Using next-generation sequencing approaches to isolate simple sequence repeat (SSR) loci in the plant sciences. Am. J. Bot..

[50] Miller A.D., Good R.T., Coleman R.A., Lancaster M.L., Weeks A.R. (2013). Microsatellite loci and the complete mitochondrial DNA sequence characterized through next generation sequencing and de novo assembly for the critically endangered orange-bellied parrot, *Neophema chrysogaster*. Mol. Biol. Rep..

[51] Zhang S.H., Luo H., Du H., Wang D.Q., Wei Q.W. (2013). Isolation and characterization of twenty-six microsatellite loci for the tetraploid fish Dabry’s sturgeon (*Acipenser dabryanus*). Conserv. Genet. Res..

[52] Feldmeyer B., Wheat C.W., Krezdorn N., Rotter B., Pfenninger M. (2011). Short read Illumina data for the *de novo* assembly of a non-model snail species transcriptome (*Radix balthica*, Basommatophora, Pulmonata), and a comparison of assembler performance. BMC Genom..

[53] Martin M. (2011). Cutadapt removes adapter sequences from high-throughput sequencing reads. EMBnet J..

[54] Jiang H., Lei R., Ding S.-W., Zhu S. (2014). Skewer: A fast and accurate adapter trimmer for next-generation sequencing paired-end reads. BMC Bioinform..

[55] Pertea G., Huang X., Liang F., Antonescu V., Sultana R., Karamycheva S., Lee Y., White J., Cheung F., Parvizi B. (2003). TIGR Gene Indices clustering tools (TGICL): A software system for fast clustering of large EST datasets. Bioinformatics.

[56] Kang S.W., Patnaik B.B., Hwang H.J., Park S.Y., Lee J.S., Han Y.S., Lee Y.S. (2015). PANM DB (Protostome DB) for the annotation of NGS data of mollusks. Korean J. Malacol..

[57] UniGene. ftp://ftp.ncbi.nih.gov/repository/UniGene/.

[58] Tatusov R.L., Fedorova N.D., Jackson J.D., Jacobs A.R., Kiryutin B., Koonin E.V., Krylov D.M., Mazumder R., Mekhedov S.L., Nikolskaya A.N. (2003). The COG database: An updated version includes eukaryotes. BMC Bioinform..

[59] Kanehisa M., Goto S., Kawashima S., Okuno Y., Hattori M. (2004). The KEGG resource for deciphering the genome. Nucleic Acids Res..

[60] Oliveros J.C. VENNY: An Interactive Tool for Comparing List with Venn Diagram. VENNY Website. http://bioinfogp.cnb.csic.es/tools/venny/index.html.

[61] Consea A., Gotz S., Garcia-Gomez J., Terol J., Talon M., Robles M. (2005). BLAST2GO: A universal tool for annotation, visualization and analysis in functional genomics research. Bioinformatics.

[62] Quevillon E., Silventoinen V., Pillai S., Harte N., Mulder N., Apweiler R., Lopez R. (2005). InterProScan: Protein domains identifier. Nucleic Acids Res..

[63] MISA-MicroSAtellite Identification Tool. http://pgrc.ipk-gatersleben.de/misa/.

[64] You F.M., Huo N., Gu Y.Q., Luo M.C., Ma Y., Hane D., Lazo G.R., Dvorak J., Anderson O.D. (2008). BatchPrimer3: A high throughput web application for PCR and sequencing primer design. BMC Bioinform..

