# Supplementary material for: Transcriptome Characterization for Non-Model Endangered Lycaenids, Protantigius superans and Spindasis takanosis, Using Illumina HiSeq 2500 Sequencing
Source: Int J Mol Sci. 2015 Dec 16;16(12):29948–70. doi: 10.3390/ijms161226213 (PMC4691156; doi:10.3390/ijms161226213)
Supplement: Supplementary file 1 [file ijms-16-26213-s001.zip › ijms-103773-Supplementary Information.pdf]

Supplementary Material: Transcriptome Characterization for Non-Model Endangered Lycaenids, *Protantigius superans* and *Spindasis takanosis*, Using Illumina HiSeq 2500 Sequencing

Bharat Bhusan Patnaik, Hee-Ju Hwang, Se Won Kang, So Young Park, Tae Hun Wang, Eun Bi Park, Jong Min Chung, Dae Kwon Song, Changmu Kim, Soonok Kim, Jae Bong Lee, Heon Cheon Jeong, Hong Seog Park, Yeon Soo Han and Yong Seok Lee

Table S1. Summary of raw read processing and assembly parameters after Illumina HiSeq 2500 sequencing of *S. takanosis* and *P. superans* transcriptome. The thinning and trimming analyses for the raw reads was done with Cutadapt program.

Data Processing Parameters	*Spindasis takanosis*	*Protantigius superans*
Raw reads		
Number of sequences	249,312,792	258,875,070
Number of bases	31,413,411,792	32,618,258,820
Total read pairs processed	124,656,396	129,437,535
Read 1 with adaptor	5,558,289 (4.5%)	5,851,738 (4.5%)
Read 2 with adaptor	7,370,351 (5.9%)	7,136,093 (5.5%)
Pairs written (passing filters)	124,656,396	129,437,535
Total base pairs processed (bp)	31,413,411,792	32,618,258,820
Read 1 (bp)	15,706,705,896	16,309,129,410
Read 2 (bp)	15,706,705,896	16,309,129,410
Total base pairs filtered (bp)	31,304,793,937	32,514,410,974
Read 1 (bp)	15,655,212,070	16,259,148,797
Read 2 (bp)	15,649,581,867	16,255,262,177
% of reads after trimming	99.65	99.68
% of reads discarded	0.35	0.32
Average length after trimming (bp)	125.6	125.6

Adaptor 1 sequence: AGATCGGAAGAGCACACGTCTGAACTCCAGTCAC; Adaptor 2 sequence: AGATCGGAAGAGCGTCGTGTAGGGAAAGAGTGTAGATCTCGGTGGTCGCCGTATCATT.

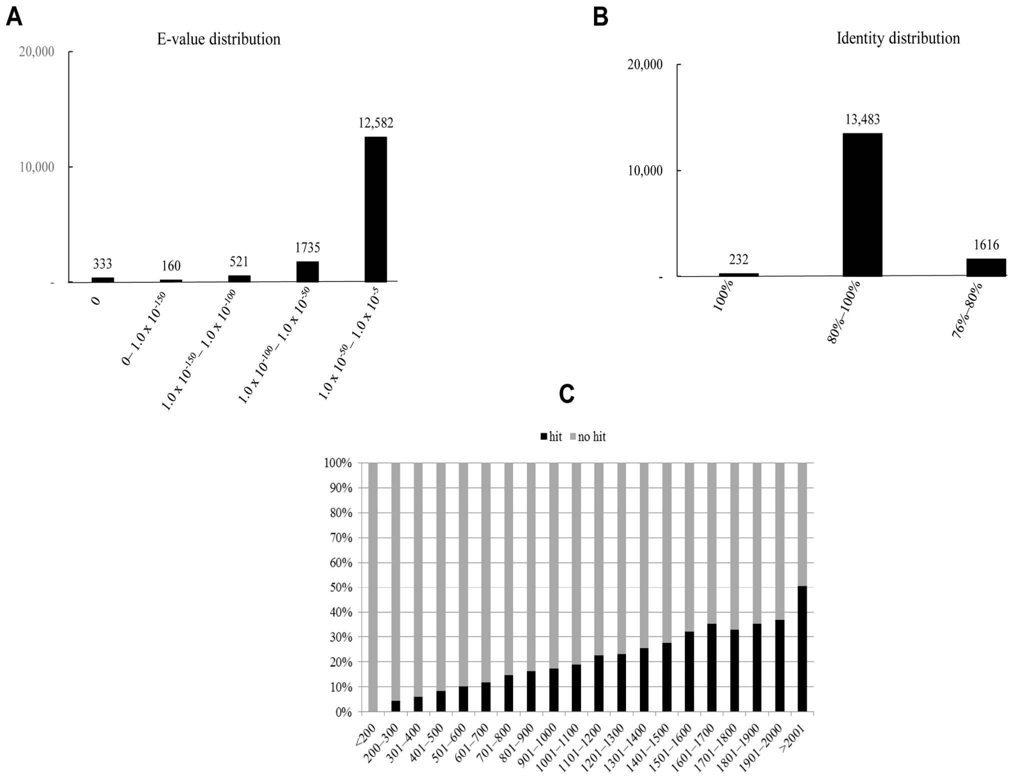

Figure S1. Characteristics of homology search of assembled sequences of *P. superans* against Unigene DB. (**A**) E-value distribution of BLAST hits for assembled sequences with a cutoff value of 1.0×10^{-5}; (**B**) Identity distribution of BLAST hits for each assembled sequence; (**C**) Comparison of the length of assembled sequences with or without hits.

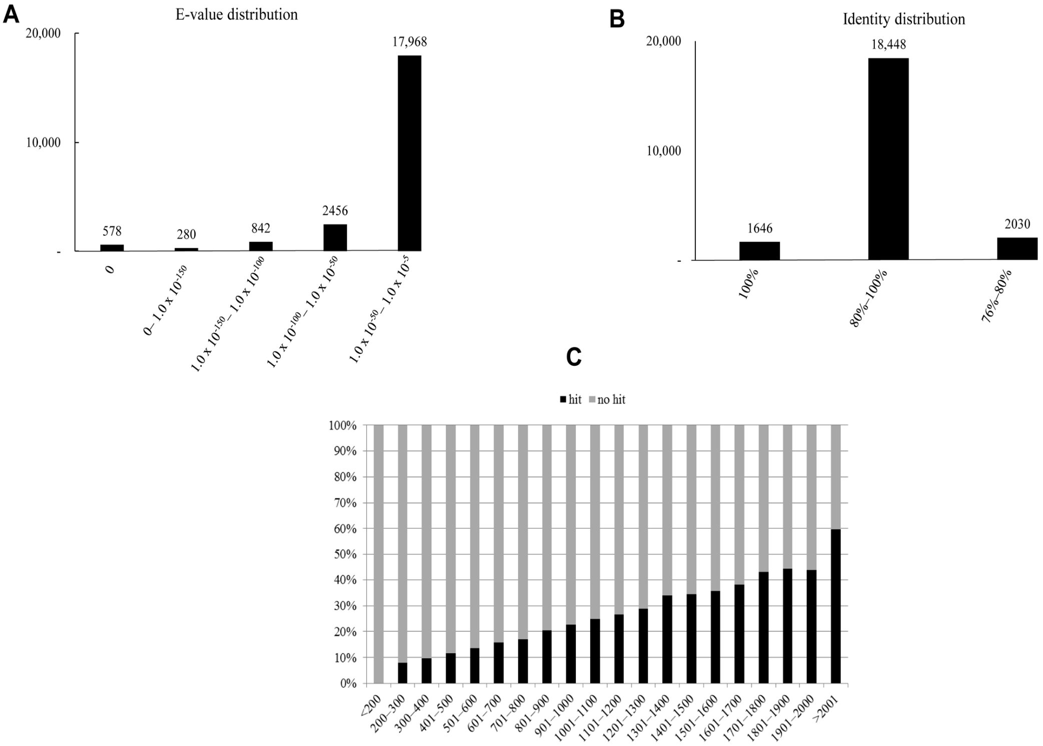

Figure S2. Characteristics of homology search of assembled sequences of *S. takanosis* against Unigene DB. (**A**) E-value distribution of BLAST hits for assembled sequences with a cutoff value of 1.0×10^{-5}; (**B**) Identity distribution of BLAST hits for each assembled sequence; (**C**) Comparison of the length of assembled sequences with or without hits.

Table S2. List of the top-hit 40 Interpro domains in *P. superans* transcriptome.

Interpro Domain	Description	Unigenes
IPR007087	Zinc finger, C2H2 domain	609
IPR001254	Serine proteases, trypsin domain	262
IPR000719	Protein kinase domain	252
IPR007588	Zinc finger, FLYWCH-type	235
IPR000477	Reverse transcriptase domain	210
IPR001680	WD40 repeat	172
IPR000504	RNA recognition motif domain	170
IPR005828	Major facilitator, sugar transporter-like family	126
IPR011701	Major facilitator superfamily	123
IPR001611	Leucine-rich repeat	121
IPR001128	Cytochrome P450 family	113
IPR005135	Endonuclease/exonuclease/phosphatase domain	108
IPR003598	Immunoglobulin subtype 2 domain	106
IPR011989	Armadillo-like helical domain	106
IPR002048	EF-hand domain	104
IPR003439	ABC transporter-like domain	99
IPR002110	Ankyrin repeat	98
IPR002018	Carboxylesterase, type B domain	93
IPR000210	BTB/POZ domain	89
IPR000618	Insect cuticle protein family	89
IPR012337	Ribonuclease H-like domain	84
IPR029526	PiggyBac transposable element-derived protein domain	76
IPR000276	G protein-coupled receptor, rhodopsin-like family	74
IPR002198	Short-chain dehydrogenase/reductase SDR family	72
IPR006578	MADF domain	71
IPR001806	Small GTPase superfamily	70
IPR001841	Zinc finger, RING-type domain	70
IPR000742	EGF-like domain	69
IPR002213	UDP-glucuronosyl/UDP-glucosyltransferase family	69
IPR020846	Major facilitator superfamily domain	66
IPR001478	PDZ domain	65
IPR001452	SH3 domain	64
IPR007110	Immunoglobulin-like domain	64
IPR011990	Tetratricopeptide-like helical domain	62
IPR000008	C2 domain	61
IPR027417	P-loop containing nucleoside triphosphate hydrolase domain	61
IPR027806	Harbinger transposase-derived nuclease domain	54
IPR006612	Zinc finger, C2CH-type domain	53
IPR013057	Amino acid transporter, transmembrane domain	53
IPR003961	Fibronectin type III domain	52

Table S3. List of top-hit 40 Interpro domains in *S. takanosis* transcriptome.

Interpro Domain	Description	Unigenes
IPR015880	Zinc finger, C2H2-like domain	425
IPR027417	P-loop containing nucleoside triphosphate hydrolase domain	283
IPR000477	Reverse transcriptase domain	264
IPR002290	Serine/threonine/dual specificity protein kinase, catalytic domain	205
IPR000504	RNA recognition motif domain	204
IPR001680	WD40 repeat	186
IPR007588	Zinc finger, FLYWCH-type domain	182
IPR011701	Major facilitator superfamily	159
IPR029058	Alpha/Beta hydrolase fold domain	157
IPR005135	Endonuclease/exonuclease/phosphatase domain	149
IPR001314	Peptidase S1A, chymotrypsin-type family	146
IPR005828	Major facilitator, sugar transporter-like family	146
IPR012337	Ribonuclease H-like domain	145
IPR011989	Armadillo-like helical domain	144
IPR002110	Ankyrin repeat	142
IPR013783	Immunoglobulin-like fold domain	140
IPR001304	C-type lectin domain	136
IPR002048	EF-hand domain	136
IPR016040	NAD(P)-binding domain	127
IPR003593	AAA+ ATPase domain	125
IPR001254	Serine proteases, trypsin domain	119
IPR001128	Cytochrome P450 family	118
IPR001478	PDZ domain	106
IPR015943	WD40/YVTN repeat-like-containing domain	100
IPR029526	PiggyBac transposable element-derived protein domain	98
IPR000276	G protein-coupled receptor, rhodopsin-like family	97
IPR011992	EF-hand domain pair domain	96
IPR029063	S-adenosyl-L-methionine-dependent methyltransferase domain	96
IPR003599	Immunoglobulin subtype domain	93
IPR003591	Leucine-rich repeat, typical subtype repeat	86
IPR002126	Cadherin domain	85
IPR003598	Immunoglobulin subtype 2 domain	85
IPR019734	Tetratricopeptide repeat	84
IPR002557	Chitin binding domain	83
IPR001452	SH3 domain	81
IPR005055	Insect odorant-binding protein A10/Ejaculatory bulb-specific protein 3 family	79
IPR002347	Glucose/ribitol dehydrogenase family	78
IPR000742	EGF-like domain	77
IPR001841	Zinc finger, RING-type domain	77
IPR012336	Thioredoxin-like fold domain	76
